# (2*E*,4*E*)-2-Cyano-5-dipropyl­amino-*N*,*N*-dimethyl­penta-2,4-dienamide

**DOI:** 10.1107/S1600536812013797

**Published:** 2012-04-06

**Authors:** Xian-Feng Zhou, Xiao-Hua Du

**Affiliations:** a State Key Laboratory Breeding Base of Green Chemistry-Synthesis Technology, Zhejiang University of Technology, Hangzhou 310014, People’s Republic of China

## Abstract

In the title compound, C_14_H_23_N_3_O, the *n*-propyl group is disordered over two orientations with an occupancy ratio of 0.778 (3):0.222 (3). In the crystal, mol­ecules are linked by pairs of weak C—H⋯O inter­actions into inversion dimers with an *R*_2_^2^(14) graph-set motif.

## Related literature

For applications of the title compound, see: Bryson *et al.* (1976 ▶). For the synthesis of *N*,*N*-dimethyl­cyano­acetamide, see: Basheer *et al.* (2007 ▶). For hydrogen-bond graph-set motifs, see Etter *et al.* (1990 ▶). For a description of the Cambridge Structural Database, see Allen (2002 ▶). For structures with disordered *n*-propyl­groups attached to CH_2_-N-CH_2_, see: Bouwman *et al.* (2000 ▶); Liu *et al.* (2005 ▶); Wang *et al.* (2009 ▶). For the extinction correction, see: Becker & Coppens (1974 ▶).
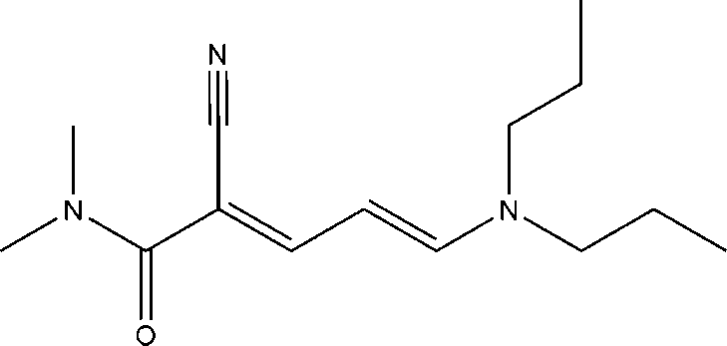


## Experimental

### 

#### Crystal data

C_14_H_23_N_3_O
*M* *_r_* = 249.35
Monoclinic, 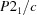

*a* = 9.0177 (6) Å
*b* = 14.0654 (9) Å
*c* = 12.9008 (8) Å
β = 111.768 (2)°
*V* = 1519.63 (17) Å^3^
*Z* = 4
Mo *K*α radiation
μ = 0.07 mm^−1^
*T* = 296 K
0.48 × 0.46 × 0.28 mm

#### Data collection

Rigaku R-AXIS RAPID/ZJUG diffractometer
Absorption correction: multi-scan (*ABSCOR*; Higashi, 1995 ▶) *T*_min_ = 0.957, *T*_max_ = 0.981
14122 measured reflections
3321 independent reflections
1412 reflections with *I* > 3σ(*I*)
*R*_int_ = 0.056

#### Refinement

*R*[*F* ^2^ > 2σ(*F* ^2^)] = 0.044
*wR*(*F* ^2^) = 0.115
*S* = 1.49
3321 reflections
171 parameters
H-atom parameters constrained
Δρ_max_ = 0.17 e Å^−3^
Δρ_min_ = −0.14 e Å^−3^

### 

Data collection: *PROCESS-AUTO* (Rigaku, 2006 ▶); cell refinement: *PROCESS-AUTO*; data reduction: *CrystalStructure* (Rigaku, 2007 ▶); program(s) used to solve structure: *SIR97* (Altomare *et al.*, 1999) ▶; program(s) used to refine structure: *JANA2006* (Petricek *et al.*, 2006 ▶); molecular graphics: *ORTEP-3* for Windows (Farrugia, 1997 ▶); software used to prepare material for publication: *WinGX* (Farrugia, 1999 ▶).

## Supplementary Material

Crystal structure: contains datablock(s) global, I. DOI: 10.1107/S1600536812013797/fb2241sup1.cif

Structure factors: contains datablock(s) I. DOI: 10.1107/S1600536812013797/fb2241Isup2.hkl

Supplementary material file. DOI: 10.1107/S1600536812013797/fb2241Isup3.cml

Additional supplementary materials:  crystallographic information; 3D view; checkCIF report

## Figures and Tables

**Table 1:** Hydrogen-bond geometry (Å, °)

*D*—H⋯*A*	*D*—H	H⋯*A*	*D*⋯*A*	*D*—H⋯*A*
C5—H5⋯O1^i^	0.93	2.46	3.375 (2)	168

Symmetry code: (i) 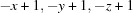.

**Fig. 1.**
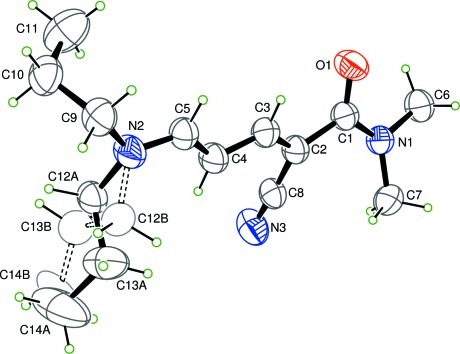
The title molecule with the atomic labelling scheme. The displacement ellipsoids are drawn at the 50% probability level.

**Fig. 2.**
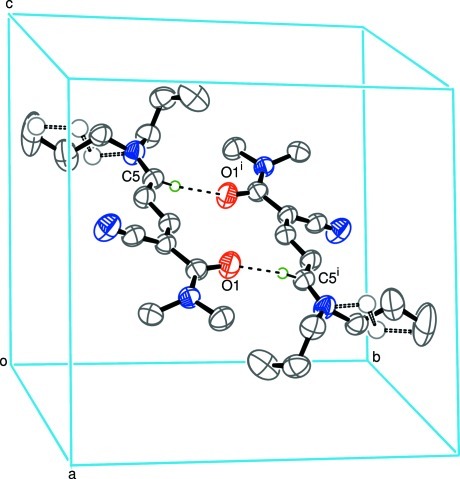
The graph set motif R22(14) showing the hydrogen bonds C5—H5···O1i. The symmetry code: (i): 1-x, 1-y, 1-z.

## supplementary crystallographic information

### Comment

The title compound is a useful intermediate for the synthesis of 2-chloro-*N*,*N*-dimethylnicotinamide derivatives, which are widely used in syntheses of pharmaceutics and pesticides (Bryson *et al.*, 1976). In order to establish conformational details of the title molecule, its crystal structure has been determined. In the crystal structure, the *n*-propyl composed of C12\C13\C14 is disordered over two positions with different occupancies that are 0.778 (3) and 0.222 (3) for the chains *A* and *B*, respectively. Both disordered chains are related approximately by a non-crystallographic symmetry plane. As shown in Fig. 1, O1 is deviated by 3.6 (4) Å from the plane composed of C1\C2\C3\C4\C5.

A search in the Cambridge Structural Database (Allen, 2002) for the structures that contain a fragment of the *n*-propyl attached to CH_2_-N-CH_2_ where N is not involved in a ring yielded 14 hits from which three of them contained a disordered *n*-propyl fragment. These three structures are FEDKEF, *i. e.**N*,*N*-bis (anthracen-9-ylmethyl)propylamine determined by Liu *et al.* (2005); HUYWAA, *i. e.* (µ2-*N*,*N*-bis((diphenylphosphino)methyl) -n-propylamine-P,P') -bis(µ2-propane-1,3-dithiolato-S,S,S',S')-decacarbonyl-tetra-iron dichloromethane solvate determined by Wang *et al.* (2009); WIGKOM, *i. e.**N*,*N*-bis(2-ethyl-5-methyl-imidazol -4-ylmethyl)aminopropane monohydrate determined by Bouwman *et al.* (2000). These examples show that the disorder of *n*-propyl chain is rather common in molecules containing such a motif as it happens to be present in the title structure (Fig. 1).

There are present only weak intermolecular interactions in the structure among which C5—H5···O1^i^ is most prominent (the symmetry code i: 1-*x*, 1-*y*, 1-*z*). As shown in Fig. 2, a centrosymmetric dimer about the crystallographic inversion centre is formed by a pair of these hydrogen bonds with the graph set motif *R*^2^_2_(14) (Etter *et al.*, 1990).

### Experimental

To a three-necked flask, 3-dipropylaminopropenal (31 g, 0.2 mol), *N*,*N*-dimethylcyanoacetamide (22.6 g, 0.2 mol) (Basheer *et al.*, 2007), anhydrous acetic acid (2.4 g, 0.04 mol), anhydrous monoethanolamine (2.44 g, 0.04 mol) and toluene (50 ml) were added. The mixture was stirred and heated to reflux for about 3 h and the generated water was separated by azeotropic distillation. After the reaction had completed, toluene was removed under vacuum. Then ethyl acetate (60 ml) was added to the residue and stirred at room temperature for 2 h. The precipitate was filtered to yield (2*E*,4*E*) -2-cyano-5-(dipropylamino)-*N*,*N*-dimethyl-2,4-pentadienamide (40 g, yield 80%). Single crystals were obtained as light yellow blocks (about 0.5–1 mm size) by slow evaporation at room temperature from a solution in petroleum ether (30–60°C).

### Refinement

The initial determination of the structural model yielded the non-disordered non-hydrogen atoms in the structure as well as the atoms C12a\C13a\C14a which form the dominant part of the disordered *n*-propyl chain. The difference electron density map has shown other maxima corresponding to the atoms of the less occupied disordered chain C12b\C13b\C14b. Moreover, the difference electron density map has also shown the hydrogen positions of all the hydrogens with exception of those pertinent to the less occupied *n*-propyl chain. The non-hydrogen atoms of both disoredered chains were related approximately by reflection through a non crystallographic mirror plane. The non-proper symmetry that related the disordered chains has been respected in the refinement which assumed that both chains were identical except for the inversion between them. Thus, the individual parameters of the atoms in the chain were refined in addition to the positional parameters of each of the disordered chain. Moreover, the occupational parameters of both chains were constrained to equal to 1.0. The described procedure infers that the positions of the methyl hydrogens of C14b which are not observable in the difference electron density maps can be biased because the methyl hydrogens pertinent to C14a and C14b need not be related by the reflection through the non-crystallographic mirror plane. The riding-atom approximation has been used for all the H-atoms in the structure: The used constraints are C_sp^2^_-H_sp^2^_=0.93, C_methyl_H_methyl_=0.96, C_methylene_-H_methylene_=0.97 Å. *U*_iso_H_sp^2^_=1.2*U*_eq_C_sp^2^_, *U*_iso_H_methylene_=1.2*U*_eq_C_methylene_, *U*_iso_H_methyl_=1.5*U*_eq_C_methyl_.

### Crystal data

**Table:**

C_14_H_23_N_3_O	*F*(000) = 544
*M**_r_* = 249.35	*D*_x_ = 1.090 Mg m^−^^3^
Monoclinic, *P*2_1_/*c*	Melting point = 343–345 K
Hall symbol: -P 2ybc	Mo *K*α radiation, λ = 0.71073 Å
*a* = 9.0177 (6) Å	Cell parameters from 7731 reflections
*b* = 14.0654 (9) Å	θ = 3.4–27.4°
*c* = 12.9008 (8) Å	µ = 0.07 mm^−^^1^
β = 111.768 (2)°	*T* = 296 K
*V* = 1519.63 (17) Å^3^	Block, yellow
*Z* = 4	0.48 × 0.46 × 0.28 mm

### Data collection

**Table:**

Rigaku R-AXIS RAPID/ZJUG diffractometer	3321 independent reflections
Radiation source: rotating anode	1412 reflections with *I* > 3σ(*I*)
Graphite monochromator	*R*_int_ = 0.056
Detector resolution: 10.00 pixels mm^-1^	θ_max_ = 27.0°, θ_min_ = 3.4°
ω scans	*h* = −11→11
Absorption correction: multi-scan (*ABSCOR*; Higashi, 1995)	*k* = −17→17
*T*_min_ = 0.957, *T*_max_ = 0.981	*l* = −16→16
14122 measured reflections	

### Refinement

**Table:**

Refinement on *F*^2^	Secondary atom site location: difference Fourier map
Least-squares matrix: full	Hydrogen site location: difference Fourier map
*R*[*F*^2^ > 2σ(*F*^2^)] = 0.044	H-atom parameters constrained
*wR*(*F*^2^) = 0.115	Weighting scheme based on measured s.u.'s *w* = 1/(σ^2^(*I*) + 0.0004*I*^2^)
*S* = 1.49	(Δ/σ)_max_ = 0.008
3321 reflections	Δρ_max_ = 0.17 e Å^−^^3^
171 parameters	Δρ_min_ = −0.14 e Å^−^^3^
0 restraints	Extinction correction: B–C type 1 Lorentzian isotropic (Becker & Coppens, 1974)
93 constraints	Extinction coefficient: 21000 (2000)
Primary atom site location: structure-invariant direct methods	

### Special details

**Table:**

Geometry. All e.s.d.'s (except the e.s.d. in the dihedral angle between two l.s. planes) are estimated using the full covariance matrix. The cell e.s.d.'s are taken into account individually in the estimation of e.s.d.'s in distances, angles and torsion angles; correlations between e.s.d.'s in cell parameters are only used when they are defined by crystal symmetry. An approximate (isotropic) treatment of cell e.s.d.'s is used for estimating e.s.d.'s involving l.s. planes.
Refinement. Refinement of *F*^2^ against ALL reflections. The weighted *R*-factor *wR* and goodness of fit *S* are based on *F*^2^, conventional *R*-factors *R* are based on *F*, with *F* set to zero for negative *F*^2^. The threshold expression of *F*^2^ > σ(*F*^2^) is used only for calculating *R*-factors(gt) *etc*. and is not relevant to the choice of reflections for refinement. *R*-factors based on *F*^2^ are statistically about twice as large as those based on *F*, and *R*- factors based on ALL data will be even larger.

### Fractional atomic coordinates and isotropic or equivalent isotropic displacement parameters (Å^2^)

**Table:**

	*x*	*y*	*z*	*U*_iso_*/*U*_eq_	Occ. (<1)
C1	0.7710 (2)	0.36154 (14)	0.45084 (13)	0.0577 (8)	
C3	0.5630 (2)	0.33029 (13)	0.52819 (12)	0.0575 (7)	
H1c3	0.53292	0.393093	0.509106	0.0689*	
C4	0.4773 (2)	0.28096 (14)	0.58200 (13)	0.0609 (8)	
H1c4	0.499509	0.217163	0.600079	0.073*	
N1	0.86144 (17)	0.32384 (10)	0.39765 (11)	0.0596 (6)	
O1	0.76404 (15)	0.44850 (10)	0.46179 (10)	0.0806 (7)	
C8	0.7432 (2)	0.20380 (15)	0.53474 (14)	0.0613 (8)	
C2	0.6858 (2)	0.29684 (13)	0.50038 (13)	0.0539 (7)	
N2	0.27560 (19)	0.29019 (11)	0.66391 (12)	0.0705 (8)	
C5	0.3609 (2)	0.32599 (13)	0.60832 (13)	0.0601 (8)	
H5	0.339131	0.388673	0.584583	0.0721*	
C7	0.8384 (2)	0.22932 (13)	0.34734 (14)	0.0763 (10)	
H1c7	0.920869	0.187559	0.393177	0.1145*	
H2c7	0.736089	0.205042	0.341428	0.1145*	
H3c7	0.843117	0.233226	0.27433	0.1145*	
C6	0.9693 (2)	0.38499 (14)	0.36768 (16)	0.0766 (10)	
H1c6	0.915903	0.408578	0.293221	0.1149*	
H2c6	1.061854	0.349311	0.371235	0.1149*	
H3c6	1.001483	0.437455	0.418793	0.1149*	
C9	0.1604 (2)	0.34923 (13)	0.68939 (16)	0.0725 (9)	
H1c9	0.060681	0.314778	0.671024	0.087*	
H2c9	0.133945	0.404351	0.640731	0.087*	
N3	0.7901 (2)	0.12920 (14)	0.56658 (14)	0.0875 (9)	
C10	0.2176 (3)	0.38138 (15)	0.80951 (16)	0.0829 (10)	
H1c10	0.136486	0.42087	0.820581	0.0995*	
H2c10	0.229544	0.326592	0.857613	0.0995*	
C11	0.3729 (3)	0.43521 (19)	0.84523 (18)	0.1130 (13)	
H1c11	0.401634	0.455953	0.921073	0.1694*	
H2c11	0.360998	0.489496	0.797697	0.1694*	
H3c11	0.455082	0.3945	0.839672	0.1694*	
C12a	0.3146 (3)	0.19427 (17)	0.7201 (2)	0.0660 (11)	0.778 (3)
H1c12a	0.289827	0.19388	0.787092	0.0792*	0.778 (3)
H2c12a	0.427849	0.181788	0.741427	0.0792*	0.778 (3)
C13a	0.2208 (6)	0.1178 (3)	0.6423 (4)	0.1043 (16)	0.778 (3)
H1c13a	0.108059	0.133368	0.615493	0.1252*	0.778 (3)
H2c13a	0.256201	0.112372	0.580105	0.1252*	0.778 (3)
C14a	0.2497 (11)	0.0232 (3)	0.7062 (8)	0.168 (4)	0.778 (3)
H2c14a	0.337182	0.030256	0.7764	0.2515*	0.778 (3)
H1c14a	0.274799	−0.02531	0.662984	0.2515*	0.778 (3)
H3c14a	0.155293	0.005554	0.719274	0.2515*	0.778 (3)
C12b	0.2241 (11)	0.1875 (6)	0.6423 (7)	0.0660 (12)	0.222 (3)
H1c12b	0.114942	0.178009	0.636844	0.0792*	0.222 (3)
H2c12b	0.233741	0.162761	0.574845	0.0792*	0.222 (3)
C13b	0.3354 (13)	0.1344 (7)	0.7415 (8)	0.1043 (18)	0.222 (3)
H1c13b	0.333099	0.16291	0.809346	0.1252*	0.222 (3)
H2c13b	0.442702	0.136583	0.74116	0.1252*	0.222 (3)
C14b	0.2797 (16)	0.0305 (7)	0.7341 (10)	0.168 (4)	0.222 (3)
H2c14b	0.200701	0.018635	0.661289	0.2515*	0.222 (3)
H1c14b	0.369246	−0.01099	0.746841	0.2515*	0.222 (3)
H3c14b	0.234411	0.019052	0.789557	0.2515*	0.222 (3)

### Atomic displacement parameters (Å^2^)

**Table:**

	*U*^11^	*U*^22^	*U*^33^	*U*^12^	*U*^13^	*U*^23^
C1	0.0571 (11)	0.0605 (13)	0.0590 (10)	−0.0015 (10)	0.0255 (9)	−0.0007 (9)
C3	0.0606 (11)	0.0574 (12)	0.0577 (10)	−0.0015 (9)	0.0257 (9)	−0.0009 (8)
C4	0.0639 (12)	0.0587 (12)	0.0708 (11)	−0.0003 (10)	0.0376 (10)	0.0002 (9)
N1	0.0639 (10)	0.0602 (10)	0.0652 (9)	−0.0069 (8)	0.0361 (8)	−0.0048 (7)
O1	0.0962 (10)	0.0573 (9)	0.1103 (10)	−0.0023 (8)	0.0641 (8)	0.0002 (7)
C8	0.0594 (12)	0.0697 (14)	0.0619 (11)	−0.0027 (10)	0.0308 (9)	0.0044 (10)
C2	0.0555 (11)	0.0552 (12)	0.0561 (10)	−0.0014 (9)	0.0265 (8)	0.0013 (8)
N2	0.0782 (11)	0.0606 (11)	0.0934 (11)	0.0013 (9)	0.0559 (9)	0.0033 (8)
C5	0.0620 (12)	0.0619 (13)	0.0623 (11)	−0.0037 (10)	0.0299 (9)	0.0011 (9)
C7	0.0923 (16)	0.0766 (15)	0.0707 (13)	−0.0047 (12)	0.0426 (12)	−0.0117 (10)
C6	0.0767 (14)	0.0827 (15)	0.0868 (14)	−0.0099 (11)	0.0494 (12)	0.0022 (11)
C9	0.0594 (12)	0.0819 (15)	0.0878 (13)	0.0070 (10)	0.0408 (10)	−0.0004 (10)
N3	0.0948 (13)	0.0784 (13)	0.1020 (13)	0.0141 (10)	0.0513 (10)	0.0235 (10)
C10	0.0889 (16)	0.0922 (16)	0.0831 (14)	0.0141 (13)	0.0498 (11)	0.0037 (11)
C11	0.0842 (16)	0.138 (2)	0.1120 (19)	0.0011 (16)	0.0305 (14)	−0.0358 (17)
C12a	0.0678 (15)	0.0697 (18)	0.0709 (16)	−0.0006 (13)	0.0378 (13)	0.0027 (13)
C13a	0.095 (2)	0.084 (2)	0.143 (3)	−0.0158 (19)	0.0541 (18)	−0.024 (2)
C14a	0.199 (5)	0.0557 (19)	0.302 (7)	−0.009 (3)	0.156 (4)	−0.002 (3)
C12b	0.064 (2)	0.0695 (18)	0.0744 (14)	−0.0016 (14)	0.0367 (13)	0.0021 (12)
C13b	0.127 (3)	0.100 (2)	0.0975 (19)	0.031 (2)	0.0550 (18)	0.0268 (17)
C14b	0.251 (9)	0.078 (2)	0.235 (4)	0.054 (4)	0.161 (4)	0.064 (3)

### Geometric parameters (Å, º)

**Table:**

C1—N1	1.354 (3)	C9—C10	1.510 (3)
C1—O1	1.236 (2)	C10—H1c10	0.97
C1—C2	1.480 (3)	C10—H2c10	0.97
C3—H1c3	0.93	C10—C11	1.506 (3)
C3—C4	1.399 (3)	C11—H1c11	0.96
C3—C2	1.368 (3)	C11—H2c11	0.96
C4—H1c4	0.93	C11—H3c11	0.96
C4—C5	1.373 (3)	C12a—H1c12a	0.97
N1—C7	1.460 (2)	C12a—H2c12a	0.97
N1—C6	1.454 (3)	C12a—C13a	1.500 (5)
C8—C2	1.417 (3)	C13a—H1c13a	0.97
C8—N3	1.149 (3)	C13a—H2c13a	0.97
N2—C5	1.330 (3)	C13a—C14a	1.536 (8)
N2—C9	1.460 (3)	C14a—H2c14a	0.96
N2—C12a	1.510 (3)	C14a—H1c14a	0.96
N2—C12b	1.512 (9)	C14a—H3c14a	0.96
C5—H5	0.93	C12b—H1c12b	0.97
C7—H1c7	0.96	C12b—H2c12b	0.97
C7—H2c7	0.96	C12b—C13b	1.500 (12)
C7—H3c7	0.96	C13b—H1c13b	0.97
C6—H1c6	0.96	C13b—H2c13b	0.97
C6—H2c6	0.96	C13b—C14b	1.536 (14)
C6—H3c6	0.96	C14b—H2c14b	0.96
C9—H1c9	0.97	C14b—H1c14b	0.96
C9—H2c9	0.97	C14b—H3c14b	0.96
			
N1—C1—O1	120.76 (19)	H1c10—C10—H2c10	105.7788
N1—C1—C2	119.01 (17)	H1c10—C10—C11	109.4707
O1—C1—C2	120.17 (18)	H2c10—C10—C11	109.4709
H1c3—C3—C4	116.2395	C10—C11—H1c11	109.4713
H1c3—C3—C2	116.2397	C10—C11—H2c11	109.4711
C4—C3—C2	127.52 (17)	C10—C11—H3c11	109.4707
C3—C4—H1c4	119.7468	H1c11—C11—H2c11	109.4715
C3—C4—C5	120.51 (17)	H1c11—C11—H3c11	109.4716
H1c4—C4—C5	119.7456	H2c11—C11—H3c11	109.4712
C1—N1—C7	124.68 (17)	N2—C12a—H1c12a	110.0628
C1—N1—C6	119.43 (16)	N2—C12a—H2c12a	109.3494
C7—N1—C6	114.79 (17)	N2—C12a—C13a	110.3 (2)
C2—C8—N3	177.4 (2)	H1c12a—C12a—H2c12a	108.1807
C1—C2—C3	120.15 (16)	H1c12a—C12a—C13a	109.4711
C1—C2—C8	121.00 (18)	H2c12a—C12a—C13a	109.4714
C3—C2—C8	118.23 (18)	C12a—C13a—H1c13a	109.4716
C5—N2—C9	120.55 (16)	C12a—C13a—H2c13a	109.4709
C5—N2—C12a	121.25 (19)	C12a—C13a—C14a	108.7 (4)
C5—N2—C12b	117.5 (5)	H1c13a—C13a—H2c13a	110.2327
C9—N2—C12a	117.31 (19)	H1c13a—C13a—C14a	109.4711
C9—N2—C12b	112.9 (4)	H2c13a—C13a—C14a	109.4713
C4—C5—N2	127.39 (17)	C13a—C14a—H2c14a	109.4714
C4—C5—H5	116.3055	C13a—C14a—H1c14a	109.4717
N2—C5—H5	116.3064	C13a—C14a—H3c14a	109.4712
N1—C7—H1c7	109.4707	H2c14a—C14a—H1c14a	109.4709
N1—C7—H2c7	109.4708	H2c14a—C14a—H3c14a	109.4711
N1—C7—H3c7	109.4714	N2—C12b—H1c12b	112.2217
H1c7—C7—H2c7	109.471	N2—C12b—H2c12b	112.6045
H1c7—C7—H3c7	109.4722	N2—C12b—C13b	104.8 (6)
H2c7—C7—H3c7	109.4713	H1c12b—C12b—H2c12b	108.1808
N1—C6—H1c6	109.4714	H1c12b—C12b—C13b	109.4711
N1—C6—H2c6	109.4711	H2c12b—C12b—C13b	109.4714
N1—C6—H3c6	109.472	C12b—C13b—H1c13b	109.4716
H1c6—C6—H2c6	109.4709	C12b—C13b—H2c13b	109.471
H1c6—C6—H3c6	109.4706	C12b—C13b—C14b	108.7 (8)
H2c6—C6—H3c6	109.4713	H1c13b—C13b—H2c13b	110.2327
N2—C9—H1c9	109.4719	H1c13b—C13b—C14b	109.4711
N2—C9—H2c9	109.4712	H2c13b—C13b—C14b	109.4713
N2—C9—C10	113.62 (15)	C13b—C14b—H2c14b	109.4714
H1c9—C9—H2c9	104.974	C13b—C14b—H1c14b	109.4718
H1c9—C9—C10	109.471	C13b—C14b—H3c14b	109.4712
H2c9—C9—C10	109.4713	H2c14b—C14b—H1c14b	109.4709
C9—C10—H1c10	109.4712	H2c14b—C14b—H3c14b	109.4711
C9—C10—H2c10	109.4722	H1c14b—C14b—H3c14b	109.4709
C9—C10—C11	112.9 (2)		

### Hydrogen-bond geometry (Å, º)

**Table:**

*D*—H···*A*	*D*—H	H···*A*	*D*···*A*	*D*—H···*A*
C5—H5···O1^i^	0.93	2.46	3.375 (2)	168

Symmetry code: (i) −*x*+1, −*y*+1, −*z*+1.
